# MicroRNAs as Emerging Therapeutic Targets Modulating the Tumor Microenvironment in Head and Neck Squamous Cell Carcinoma

**DOI:** 10.3390/ijms262110794

**Published:** 2025-11-06

**Authors:** Roxana Daniela Brata, Lavinia Marcut, Alina Cristina Barb, Alexia Manole, Alexandru Ciolofan, Cristina Stefania Dumitru, Flavia Zara, Raul Patrascu

**Affiliations:** 1 Department of Medical Disciplines, Faculty of Medicine and Pharmacy, University of Oradea, 410073 Oradea, Romania; brata.roxanadaniela@didactic.uoradea.ro; 2 Department of Surgical Sciences, Faculty of Medicine and Pharmacy, University of Oradea, 410073 Oradea, Romania; lmarcut@uoradea.ro; 3 Department II of Microscopic Morphology, Discipline of Histology, Victor Babes University of Medicine and Pharmacy Timisoara, 300041 Timisoara, Romania; toma.alina@umft.ro (A.C.B.); flavia.zara@umft.ro (F.Z.); 4 Faculty of Medicine and Pharmacy, University of Oradea, 410087 Oradea, Romania; manole.alexia@student.uoradea.ro; 5 Department IX, Discipline of Surgical Semiology I, Victor Babes University of Medicine and Pharmacy Timisoara, 300041 Timisoara, Romania; ciolofan.alexandru@umft.ro; 6 Department of Functional Sciences, Victor Babes University of Medicine and Pharmacy, 300041 Timisoara, Romania; patrascu.raul@umft.ro

**Keywords:** microRNA, head and neck cancer, tumor microenvironment, nanotherapy, immune modulation, therapeutic targets, exosomes

## Abstract

Head and neck squamous cell carcinoma (HNSCC) remains one of the most aggressive solid tumors, characterized by marked molecular heterogeneity and a complex tumor microenvironment (TME). Recent evidence highlights the pivotal role of microRNAs (miRNAs) in regulating tumor progression, immune evasion, angiogenesis, and stromal remodeling. This review synthesizes current insights into miRNA-mediated molecular pathways that modulate the TME in HNSCC and discusses emerging therapeutic strategies, including nanocarrier- and exosome-based miRNA delivery systems, targeting these molecules. Key miRNAs, including miR-21, miR-146a, and miR-221, orchestrate bidirectional signaling between cancer cells, fibroblasts, and immune infiltrates, thereby shaping tumor aggressiveness and therapy resistance. Advances in nanotechnology have facilitated the development of miRNA-based therapeutics—such as mimics, antagomiRs, and exosome-mediated systems—capable of restoring physiological expression patterns and reprogramming the TME toward an anti-tumor state. However, clinical translation remains hindered by challenges in targeted delivery, molecular stability, and tumor heterogeneity. By integrating molecular and translational perspectives, this review underscores how miRNA-targeting strategies may evolve into a new generation of precision therapies, bridging the gap between molecular oncology and personalized treatment of head and neck cancer.

## 1. Introduction

Head and neck squamous cell carcinoma (HNSCC) ranks among the most prevalent and aggressive malignancies worldwide, with approximately 890,000 new cases and over 450,000 deaths annually [1]. Despite advances in surgery, radiotherapy, and targeted therapies, the 5-year survival rate remains stagnant, largely due to late diagnosis, locoregional recurrence, and resistance to conventional treatments [2]. These poor outcomes underscore the critical role of the tumor microenvironment (TME) in dictating tumor progression and therapeutic response.

The TME comprises a heterogeneous network of cancer-associated fibroblasts, immune and endothelial cells, extracellular matrix components, and soluble mediators such as cytokines and growth factors. This dynamic interplay shapes tumor growth, angiogenesis, invasion, and immune evasion [3]. Emerging evidence suggests that molecular cross-communication between tumor cells and their microenvironment is mediated by non-coding RNAs, particularly microRNAs (miRNAs), which act as pivotal regulators of gene expression at the post-transcriptional level [4].

MicroRNAs are small non-coding RNA molecules of approximately 18–25 nucleotides that modulate the expression of target messenger RNAs (mRNAs) through translational repression or degradation. Their deregulated expression has been documented in virtually all stages of HNSCC development, from epithelial dysplasia to invasive carcinoma. Aberrant miRNA profiles contribute to malignant transformation, metastasis, angiogenesis, and immune escape [5].

Several key miRNAs, including miR-21, miR-146a, and miR-221, have been shown to orchestrate tumor–stroma interactions, thereby influencing tumor proliferation and the recruitment of immune and endothelial cells [6]. For instance, miR-21 upregulates matrix metalloproteinases and downregulates PTEN, promoting extracellular matrix remodeling and invasiveness [7]. Conversely, tumor-suppressive miRNAs such as miR-34a and miR-200c act as negative regulators of epithelial–mesenchymal transition (EMT) and angiogenesis [8].

Recent advances in nanomedicine and molecular oncology have paved the way for therapeutic modulation of miRNAs [9]. Synthetic mimics and inhibitors, as well as exosome-based delivery systems, have demonstrated the potential to restore normal miRNA expression and reprogram the TME toward an anti-tumor state [10]. However, challenges remain regarding specificity, stability, and potential off-target effects.

This review aims to summarize the current knowledge on miRNA-mediated regulation of the tumor microenvironment in HNSCC and to discuss emerging therapeutic strategies targeting these molecular pathways. By integrating molecular, histopathological, and translational perspectives, we highlight how miRNAs represent promising tools for the development of personalized, multi-targeted interventions in head and neck oncology.

## 2. Molecular Crosstalk Between microRNAs and the Tumor Microenvironment

The TME of HNSCC represents a dynamic and highly interactive ecosystem, in which tumor cells coexist with fibroblasts, immune cells, endothelial cells, and extracellular matrix components. This intricate network not only sustains cancer cell proliferation and invasion but also contributes to therapeutic resistance and immune evasion [11]. Within this complex milieu, miRNAs function as crucial molecular mediators that fine-tune intercellular communication. Through paracrine and autocrine signaling, miRNAs regulate transcriptional programs in both tumor and stromal compartments, thereby influencing processes such as epithelial–mesenchymal transition (EMT), angiogenesis, and inflammation [5,12]. Accumulating evidence suggests that tumor-derived miRNAs can be secreted via exosomes, modulating the phenotype of surrounding cells and creating a pro-tumorigenic microenvironment [13]. Conversely, stromal or immune cell–derived miRNAs may exert tumor-suppressive functions by restoring anti-inflammatory and anti-angiogenic balance [14]. Understanding this bidirectional crosstalk is essential for identifying molecular targets amenable to therapeutic modulation and for developing novel strategies that disrupt the supportive role of the TME in HNSCC progression.

### 2.1. miRNA–Cancer Cell Interactions

In HNSCC, deregulated expression of specific microRNAs reorganizes intracellular signaling cascades that regulate proliferation, apoptosis, and invasiveness. Among the most consistently upregulated molecules, miR-21 functions as a central oncogenic driver by targeting tumor suppressors such as PTEN and PDCD4, leading to activation of the PI3K/AKT and MAPK pathways that promote cell survival and chemoresistance. Elevated miR-21 levels have been associated with increased proliferation, migration, and cisplatin resistance in HNSCC cells through suppression of PTEN expression [15]. Moreover, inverse correlations between miR-21 and PDCD4 expression have been observed in nasopharyngeal carcinoma, supporting its tumor-promoting role [16]. miR-21 carried in extracellular vesicles can also downregulate TPM1, PDCD4, and PTEN in recipient cells, enhancing matrix metalloproteinase activity and extracellular matrix degradation [17].

Similarly, miR-31 is frequently overexpressed in HNSCC and promotes tumor cell migration and invasion. It suppresses FIH-1 (Factor Inhibiting HIF-1), leading to constitutive activation of HIF signaling and a hypoxia-adaptive phenotype that supports tumor persistence under stress conditions [18]. In oral squamous cell carcinoma, miR-31 has been shown to modulate RhoA and ERK/MMP9 signaling pathways, contributing to metastatic potential [19].

Conversely, several miRNAs act as tumor suppressors and are downregulated during malignant transformation. miR-34a, transcriptionally regulated by p53, inhibits epithelial–mesenchymal transition (EMT) by repressing Snail and ZEB1, thereby limiting metastatic potential. In HNSCC, reduced miR-34a levels are frequently associated with TP53 mutation or loss, and restoration of miR-34a can inhibit MET-driven oncogenic signaling [20]. Similarly, miR-200c maintains epithelial identity by targeting ZEB1 and ZEB2 and suppressing EMT, thus preventing tumor dissemination [21]. Downregulation of miR-200c in HNSCC is correlated with enhanced invasiveness and the maintenance of cancer stem cell phenotypes through BMI1 modulation [22].

Another important tumor suppressor, miR-375, is markedly underexpressed in HNSCC. Profiling studies have identified miR-375 among the most consistently reduced miRNAs in tumor tissue, with functional assays confirming its inhibitory effects on proliferation and clonogenic growth through regulation of JAK2 and IGF1R signaling [23,24].

Collectively, these findings highlight that the deregulated miRNA network in HNSCC acts not merely as a passive byproduct of tumor progression but as an active molecular orchestrator of carcinogenic behavior. Altered intracellular miRNA profiles influence not only signal transduction but also the composition of extracellular vesicles, linking intracellular oncogenic reprogramming to tumor microenvironment remodeling. For instance, exosomal miR-21 can downregulate PDCD4, TPM1, and PTEN in neighboring cells, promoting tumor invasion and immune evasion [17]. This bidirectional communication between cancer cells and their microenvironment underscores the systemic impact of miRNA dysregulation, which will be explored in subsequent sections.

The oncogenic and tumor-suppressive miRNAs involved in head and neck squamous cell carcinoma orchestrate a broad range of molecular events that sustain tumor proliferation, invasion, and resistance. Table 1 summarizes the key microRNAs most frequently reported in HNSCC, their principal targets, and the signaling pathways they regulate within tumor cells. This overview highlights the dualistic roles of miRNAs in maintaining the molecular equilibrium between oncogenic activation and tumor suppression.

### 2.2. miRNA-Mediated Immune Modulation

The immune landscape of HNSCC is profoundly shaped by dysregulated microRNAs that influence both innate and adaptive responses. Tumor- and stroma-derived miRNAs can act as immunomodulatory signals, suppressing antigen presentation and cytokine output and thereby promoting immune evasion. For example, miR-146a attenuates pro-inflammatory signaling by directly targeting IRAK1 and TRAF6, key adapters in TLR/NF-κB pathways—mechanistically linked to reduced secretion of IL-6 and TNF-α in epithelial and myeloid systems, a paradigm broadly applicable to tumor contexts [25,26]. In HNSCC, immune-related miRNA shifts are evident in tissue, where an miR-146a/miR-155 signature correlates with immune-cell-related transcripts, consistent with a remodeled tumor immune microenvironment [27].

Likewise, miR-155—often elevated in HNSCC—has dual roles: it can enhance T-cell activation in physiological contexts, yet chronic upregulation within tumors is associated with protumoral immune remodeling (e.g., macrophage polarization and T-cell dysfunction) and carries prognostic value in HNSCC [28,29]. Controversies and context dependence. Although miR-146a and miR-155 are frequently labeled as tumor-suppressive and oncogenic, respectively, both display context-dependent and sometimes opposing effects. For miR-146a, NF-κB dampening through IRAK1/TRAF6 targeting can reduce inflammatory cytokines and support anti-tumor immunity, yet in specific inflammatory milieus negative-feedback regulation may also favor tumor persistence by blunting acute immune activation [25,26]. For miR-155, transient expression in effector T cells can enhance anti-tumor responses, whereas sustained overexpression in TAMs is linked to pro-tumoral immune remodeling (M2 polarization, T-cell dysfunction) and adverse prognosis in HNSCC [28,29]. Such discrepancies likely arise from cell-type specificity (T cells vs. macrophage subsets), cytokine context, HPV status, and experimental design, underscoring the need for standardized models and validation across independent HNSCC cohorts [27,28,29,30].

Conversely, downregulation of immune-stimulatory miRNAs diminishes cytotoxic responses and interferon-linked programs. In HNSCC, miR-34a is frequently reduced; restoring miR-34a activity not only targets oncogenic MET but also associates with an antitumor immune milieu (higher Th1 and naïve CD8^+^ T-cell frequencies) and fewer PD-L1^+^ tumor-associated macrophages [31]. The miR-125 family also regulates immune function; experimental gain of miR-125b-5p enhances IFN-γ^+^ CD8^+^ T cells and limits Tregs, implying that loss of miR-125b undermines interferon-driven cytotoxicity (with supportive HNSCC tumor-suppressor evidence for miR-125b-1 loss) [32]. These alterations exemplify how miRNA-directed reprogramming of immune cells contributes to immune escape in HNSCC [30].

Altered intracellular miRNA networks also reshape the extracellular vesicle cargo, exporting oncomiRs that propagate immune suppression and pro-tumor signaling systemically. TAM-derived EV miR-21-5p promotes pro-angiogenic signaling in HNSCC tumor cells, underscoring EV-mediated TME crosstalk [33,34]. Collectively, these data suggest that therapeutic normalization of select miRNAs—either by restoring suppressive miRNAs (e.g., miR-34a/miR-125b) or inhibiting oncomiRs (e.g., miR-155/miR-146a context-dependently)—could reinvigorate immune surveillance and improve responses to immunotherapy in HNSCC [35].

The immune-modulatory functions of microRNAs extend beyond intrinsic tumor signaling, shaping the recruitment and activation of macrophages, lymphocytes, and regulatory T cells within the tumor microenvironment. As shown in Table 2, specific miRNAs such as miR-146a and miR-155 act as molecular checkpoints that determine the balance between pro- and anti-tumor immunity, influencing cytokine secretion and the polarization of immune subsets in HNSCC.

### 2.3. miRNA Regulation of Angiogenesis and Stromal Remodeling

Angiogenesis and stromal remodeling are indispensable for sustaining tumor growth and metastatic spread in HNSCC, and both processes are tightly controlled by miRNA-mediated regulation. Among the best-characterized pro-angiogenic miRNAs, miR-210 is robustly induced under hypoxic conditions via HIF-1α activation and promotes endothelial cell survival, migration, and tube formation by repressing Ephrin-A3 (EFNA3) and mitochondrial regulators ISCU1/2. This adaptation enhances vascular density within hypoxic tumor regions, supporting metabolic flexibility and survival under low-oxygen stress. In endothelial cells, blocking miR-210 impairs tubulogenesis and chemotaxis, and luciferase assays validate EFNA3 as a direct miR-210 target [36,37].

Furthermore, miR-21 also contributes to neovascularization: by targeting PTEN and activating PI3K/AKT signaling in endothelial and stromal cells, miR-21 promotes angiogenic switching, an effect complementary to its known oncogenic roles in tumor cells. Indeed, in various tumor models, miR-21 enhances vascularization and is considered a proangiogenic miRNA [37,38,39]. It should be noted that, although direct studies on HNSCC stromal/endothelial cells are more limited, the mechanism is consistent across all cancer types.

In contrast, miR-126 and miR-218 function as protective, anti-angiogenic miRNAs. miR-126 is known to maintain vascular integrity and to suppress pathological angiogenesis by negatively regulating VEGF signaling, PI3K/AKT pathways, and influencing endothelial cell responsiveness [40,41]. Its downregulation in tumors has been linked to aberrant microvasculature and leaky vessels, conditions that facilitate invasion and metastasis [42,43]. Regarding miR-218, though direct evidence in HNSCC is more limited, it is known from other cancer contexts to suppress angiogenesis via negative regulation of Slit-Robo signaling axes (thus constraining vessel sprouting and aberrant branching).

Beyond endothelial regulation, miRNAs also modulate cancer-associated fibroblasts (CAFs) and extracellular matrix (ECM) remodeling. Members of the miR-29 family suppress collagen and fibronectin gene expression (e.g., COL1A1, COL3A1, FN1), thereby limiting excessive desmoplasia characteristic of aggressive tumor stroma. Overexpression of miR-29 in multiple tumor models reduces angiogenesis by targeting VEGF, PDGF, and MMP-2 mRNAs [38]. In HNSCC specifically, miR-29c-3p has been shown to impair angiogenesis, proliferation, migration, and invasion by targeting C1QTNF6, pointing to stromal and vascular regulatory roles in this cancer subtype [44].

Conversely, miR-199a and miR-214, when dysregulated in stromal cells, can enhance fibroblast activation and promote ECM degradation via upregulation of matrix metalloproteinases (MMPs), reinforcing motility of cancer cells. Although the evidence in HNSCC is less mature, studies in other tumor types have implicated miR-199a/214 clusters in modulating TGF-β, ECM, and fibroblast phenotypes [38,45].

Together, these observations emphasize that miRNAs act as master regulators of stromal plasticity, coordinating a balance between vascular support, ECM integrity, and fibroblast activation. In HNSCC, the dysregulation of these miRNA networks likely contributes to malformed vasculature, desmoplastic stroma, and enhanced invasive potential. Targeting miRNA-dependent networks—by restoring anti-angiogenic miRNAs (e.g., miR-126, miR-29) or inhibiting pro-angiogenic/stromal miRNAs (e.g., miR-210, miR-21, miR-199a/214)—could normalize the tumor stroma, restrict invasion, and sensitize tumors to therapy.

The regulation of angiogenesis and stromal dynamics by microRNAs represents a pivotal mechanism through which tumor progression and metastasis are sustained. Table 3 outlines the principal miRNAs implicated in vascular remodeling and fibroblast activation in HNSCC, distinguishing between pro-angiogenic and anti-angiogenic molecules that collectively define the vascular phenotype of the tumor microenvironment.

**Figure 1:**
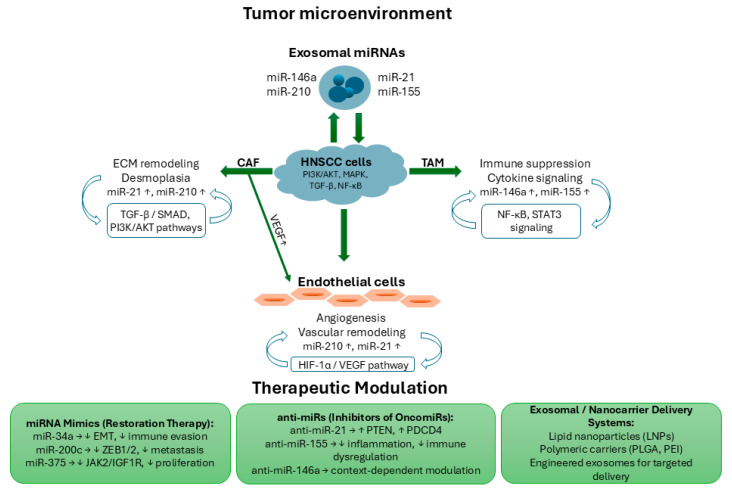
Schematic representation of miRNA-mediated crosstalk within the tumor microenvironment of HNSCC and potential therapeutic modulation strategies. Exosomal miRNAs (miR-21, miR-146a, miR-155, and miR-210) are released by stromal and immune cells, mediating bidirectional communication with HNSCC cells. Upregulated miRNAs promote extracellular matrix (ECM) remodeling and desmoplasia in cancer-associated fibroblasts (CAFs) via TGF-β/SMAD and PI3K/AKT pathways, immune suppression and cytokine signaling in tumor-associated macrophages (TAMs) through NF-κB and STAT3 pathways, and angiogenesis in endothelial cells through the HIF-1α/VEGF pathway. Central HNSCC cells are regulated by multiple signaling axes (PI3K/AKT, MAPK, TGF-β, and NF-κB), while exosomal miRNAs reinforce intercellular signaling across the tumor microenvironment. Therapeutic approaches include miRNA mimics (miR-34a, miR-200c, miR-375) to restore tumor-suppressor activity, anti-miRs (anti-miR-21, anti-miR-155, anti-miR-146a) to inhibit oncogenic miRNAs, and exosomal/nanocarrier delivery systems (LNPs, PLGA, PEI) for targeted administration.

The intricate crosstalk between tumor cells and their surrounding microenvironment is largely orchestrated by dysregulated miRNAs. These small non-coding RNAs mediate intercellular communication via exosomal transfer, modulating stromal remodeling, angiogenesis, and immune evasion. Figure 1 schematically summarizes the major miRNA-driven interactions within the tumor microenvironment of HNSCC and highlights potential therapeutic modulation strategies aimed at restoring physiological signaling balance.

Beyond their individual target networks, miRNAs frequently compete for shared mRNA binding sites within the same tumor microenvironment (TME), shaping post-transcriptional regulation in a combinatorial and context-dependent manner. In HNSCC, miR-21, miR-155, and miR-146a often converge on common targets such as PTEN, TRAF6, and STAT3, generating dynamic feedback between oncogenic and immunomodulatory pathways [27,28,36,38]. For instance, overexpression of miR-21 can repress PTEN and indirectly enhance miR-155 expression via the PI3K/AKT/NF-κB axis, while miR-146a counterbalances this effect by downregulating IRAK1/TRAF6, acting as a negative feedback regulator of inflammation.

Such miRNA–miRNA regulatory feedback loops exemplify an additional layer of complexity in HNSCC biology: oncogenic miRNAs can reinforce tumor-promoting circuits, whereas tumor-suppressive miRNAs attempt to restore homeostasis. Understanding these competing and cooperative interactions is critical for designing multi-miRNA therapeutic approaches, where simultaneous modulation of functionally linked miRNAs may yield superior antitumor effects compared to single-miRNA targeting.

### 2.4. HPV-Positive Versus HPV-Negative HNSCC: miRNA Signatures and Tumor Microenvironmental Differences

The human papillomavirus (HPV) infection introduces a distinct molecular and immunologic landscape in head and neck squamous cell carcinoma (HNSCC), reflected in specific microRNA (miRNA) expression signatures and tumor microenvironment (TME) interactions. HPV-positive tumors are characterized by increased expression of miR-9, miR-34a, and miR-363, which modulate immune checkpoints and enhance CD8^+^ T-cell infiltration, contributing to improved prognosis and treatment response. Conversely, HPV-negative tumors frequently show upregulation of miR-21, miR-155, and miR-31, correlating with enhanced epithelial–mesenchymal transition (EMT), angiogenesis, and resistance to apoptosis [40,41,42,43].

Viral oncoproteins E6 and E7 directly influence host miRNA transcription through p53 and Rb pathway inactivation, altering cell-cycle regulation and immune signaling. For example, E6-mediated p53 degradation suppresses miR-34a, while E7 promotes miR-20a and miR-27b expression, facilitating immune evasion and cell proliferation [41,44].

In the TME, HPV-positive HNSCCs typically exhibit higher immune cell infiltration (particularly cytotoxic T lymphocytes and M1 macrophages) and reduced stromal fibrosis, aligning with a more “inflamed” phenotype and enhanced radiosensitivity. In contrast, HPV-negative tumors are enriched in cancer-associated fibroblasts (CAFs), immunosuppressive M2 macrophages, and exhibit stronger activation of TGF-β–dependent stromal signaling [42,45].

These molecular and microenvironmental contrasts underscore the importance of HPV-based patient stratification in miRNA biomarker studies and therapeutic designs. Integrating HPV status into miRNA profiling could improve predictive accuracy and guide the development of personalized miRNA-targeted therapies for distinct HNSCC subtypes [40,41,42,43,44,45].

## 3. Therapeutic Modulation of microRNAs in Head and Neck Cancer

In miRNA replacement therapy, synthetic miRNA mimics or agomiRs are delivered to restore the function of miRNAs lost or suppressed in cancer cells or stromal compartments. Because a miRNA mimic can simultaneously regulate multiple targets, this approach can modulate entire oncogenic networks rather than a single gene. For example, in preclinical models, restoring miR-34 (a frequently suppressed miRNA in many tumors) suppresses proliferation, induces apoptosis, and sensitizes tumors to chemotherapy. Clinical translation of this concept was attempted in the miR-34a mimic MRX34, though the trial was prematurely terminated due to immune-related adverse events [46]. In HNSCC specifically, modulation of tumor-suppressive miRNAs such as miR-200c has been explored, including using exosomal delivery of miR-200c to revert epithelial–mesenchymal transition and enhance therapy sensitivity [47]. These strategies are promising, although delivery, off-target effects, and immunogenicity remain major challenges.

miRNA inhibition therapy targets oncogenic miRNAs (oncomiRs) to blunt their pro-tumor effects. Common modalities include antagomiRs (chemically modified antisense oligonucleotides), miRNA sponges (decoy sequences with multiple binding sites), and emerging small molecule inhibitors that modulate miRNA biogenesis or binding. Therapeutic inhibition of miR-21 (e.g., antagomiRs/LNA) restores PTEN/PDCD4 signaling and suppresses growth in preclinical models, with supportive evidence in HNSCC (CAF transition) and other solid tumors [48]. Recent reviews also examine small molecules that disrupt miRNA processing or miRNA-target interaction, offering an orthogonal route to suppress overactive miRNAs without using large oligonucleotides [49].

A critical barrier in miRNA therapy is efficient, safe, and cell-specific delivery. To that end, nanoparticles, lipid-based carriers (liposomes, lipid nanoparticles, LNPs), polymeric vectors, and exosomes/extracellular vesicles are increasingly used to protect miRNAs from nuclease degradation, reduce off-target distribution, and enhance cellular uptake. Reviews detail advances in viral and non-viral carriers tailored for miRNA delivery in cancer therapy [50]. In HNSCC and other solid tumors, nanomedicine-based strategies are under development to co-deliver miRNAs alongside chemotherapeutics or immunomodulators, aiming to remodel the TME and improve therapeutic index [51].

While miRNA-based therapies hold great promise, significant challenges impede their clinical translation. Key obstacles include off-target effects, immunogenicity, toxicity, dose optimization, targeting specificity, and scale-up manufacturing. The first clinical forays, such as MRX34 (miR-34a mimic), were discontinued partly due to immune-related adverse events, underscoring the delicate balance needed between potency and safety [46]. Nonetheless, progress is ongoing: recent reviews catalog active or completed miRNA clinical trials across cancers, highlighting both successes and lessons learned [52,53]. Meta-analyses and systematic reviews also explore ncRNA therapeutics more broadly, charting the evolving pipeline of miRNA-based cancer treatments [54].

In summary, therapeutic modulation of miRNAs in HNSCC is a promising frontier—with both mimicry and inhibition strategies under exploration—but its success hinges on overcoming delivery, specificity, and safety hurdles to realize their translational potential.

### 3.1. miRNA Mimics and Inhibitors

Synthetic modulation of microRNAs represents one of the most direct strategies for restoring molecular balance in HNSCC. miRNA mimics are double-stranded RNA analogs that emulate the activity of tumor-suppressive miRNAs whose expression is lost or suppressed. After cellular uptake, these mimics are incorporated into the RNA-induced silencing complex (RISC), guiding it to complementary mRNA targets to suppress translation or promote degradation. For example, synthetic miR-34a mimics have shown potent anti-tumor activity in preclinical models: they inhibit epithelial–mesenchymal transition (EMT) and induce apoptosis by targeting BCL2 and MET, among other oncogenes [55]. In multiple myeloma models, lipid-formulated miR-34a mimics reduced tumor growth without systemic toxicity, demonstrating proof of principle for miR-34a as a therapeutic agent [55]. In prostate cancer models, a chemically modified miR-34a variant (FM-miR-34a) reduced expression of c-Myc, MET, and androgen receptor with enhanced specificity and stability [56].

Similarly, mimics of miR-375 (a miRNA often downregulated in head and neck cancers) are envisioned to suppress proliferative signaling via repression of JAK2 or IGF1R. While direct in vivo HNSCC data on miR-375 mimics are more limited, the concept is supported by functional studies in other tumor types.

In contrast, miRNA inhibitors (also known as antagomiRs, anti-miRs, or miRNA sponges) are chemically modified single-stranded oligonucleotides designed to neutralize oncogenic miRNAs. These inhibitors bind to the target miRNA, preventing its interaction with mRNA targets. For example, anti-miR-21 (often in LNA-modified form) has been used in various cancer cell models to restore tumor suppressor expression (such as PTEN and PDCD4) and reduce cellular motility and invasiveness [57]. In colon cancer, LNA-anti-miR-21 reduced invasion, induced apoptosis, and decreased viability in vitro [58]. In melanoma mouse models, treatment with LNA-anti-miR-21 decreased tumor growth in vivo [59].

To improve stability, specificity, and in vivo half-life, modifications such as locked nucleic acid (LNA) residues, phosphorothioate backbones, and cholesterol conjugation have been adopted. These chemical enhancements make the inhibitors more resistant to nucleases and improve their circulation time [60].

Despite their promise, miRNA mimics and inhibitors face significant limitations: rapid nuclease degradation in systemic circulation, off-target binding effects, suboptimal tumor penetration, and potential immune stimulation. These issues have spurred the development of advanced delivery systems (e.g., nanoparticles, targeted carriers) that allow localized release and better pharmacokinetics, which are discussed in the following section.

Advances in synthetic biology have enabled the design of miRNA mimics and inhibitors capable of restoring or silencing specific molecular pathways deregulated in HNSCC. Table 4 compiles representative examples of these therapeutic strategies, emphasizing their molecular targets, preclinical outcomes, and mechanistic impact across different tumor models.

A critical limitation of single-miRNA inhibition is the potential activation of compensatory regulatory networks that restore oncogenic signaling. In HNSCC, suppression of miR-21 or miR-155 can trigger compensatory upregulation of other oncomiRs or long non-coding RNAs (lncRNAs) that act as competing endogenous RNAs (ceRNAs), sequestering tumor-suppressive miRNAs and thereby reactivating growth-promoting pathways. For example, lncRNA HOTAIR and MALAT1 were both upregulated in HNSCC, sponge miR-34a and miR-200c, counteracting tumor-suppressive effects on the EMT and PI3K/AKT axes [50,59,61,62,63,64]. Similarly, blockade of miR-21 in preclinical models led to increased expression of miR-10b and miR-31, which target overlapping mRNAs involved in invasion and metastasis [50,62].

These findings suggest that network-level redundancy between miRNAs and lncRNAs contributes to adaptive resistance against single-miRNA therapies. Consequently, rationally designed multi-miRNA or miRNA–lncRNA combinatorial strategies may offer superior and more durable inhibition of oncogenic signaling in HNSCC.

### 3.2. Delivery Systems and Nanocarriers

The clinical success of miRNA-based therapies hinges critically on delivery systems that can preserve molecular integrity, enable selective targeting, and promote sustained intracellular release. Naked RNA molecules are highly vulnerable to nuclease degradation, rapid renal clearance, and uptake by the mononuclear phagocyte system; therefore, advanced carriers are engineered to surmount these obstacles.

Lipid-based nanoparticles (LNPs) are among the most advanced and widely used platforms for RNA delivery. They encapsulate miRNA mimics or inhibitors within biocompatible lipid bilayers, shielding them from enzymatic degradation while facilitating cellular internalization via endocytosis. Ionizable or cationic lipids are key components: they interact electrostatically with nucleic acids at formulation pH, help condense the cargo, and, upon endosome acidification, adopt cationic forms that disrupt endosomal membranes to enable endosomal escape and cytoplasmic release. Reviews detailed the structural optimization of LNPs, biodistribution, immunogenicity, and design strategies to push delivery beyond typical hepatic tropism [50,61].

Polymeric nanoparticles provide an alternative strategy, using polymers such as polyethyleneimine (PEI), poly (lactic-*co*-glycolic acid) (PLGA), or chitosan. These materials allow customizable surface chemistry to conjugate targeting ligands (e.g., antibodies, peptides) or stealth moieties (e.g., PEG), improving tissue selectivity and circulation stability. In heterogeneous tumors like HNSCC, this adaptability is valuable for overcoming stromal barriers. Nanoparticle reviews highlight how polymeric carriers can modulate release kinetics, reduce off-target uptake, and facilitate co-delivery of miRNAs with chemotherapeutics [62,63].

Exosome-based systems, derived from natural extracellular vesicles (EVs), are gaining prominence as nearly ideal miRNA carriers. Because exosomes are endogenously secreted, they possess intrinsic biocompatibility, low immunogenicity, and inherent tropism for recipient cells. Engineered exosomes loaded with miRNA mimics have demonstrated efficacy in modulating tumor or stromal signaling in preclinical models. For example, MSC-derived exosomes carrying miRNAs have been used to restore tumor-suppressive signaling while mitigating immune activation [64,65]. However, exosome strategies face challenges in scalable isolation, consistent loading efficiency, in vivo stability, and regulatory standardization [65,66].

The selective enrichment of specific miRNAs (such as miR-21 and miR-210) in exosomes from HNSCC cells is an active, regulated process rather than a passive one. Experimental studies indicate that short sequence motifs (EXO-motifs) facilitate interaction with RNA-binding proteins (RBPs) such as hnRNPA2B1 and YBX1, which mediate miRNA sorting into multivesicular bodies [64,65,66]. Moreover, cellular stress and hypoxia, commonly observed in the HNSCC microenvironment, promote selective export of miR-210 through HIF-1α-dependent mechanisms, linking stress adaptation with exosomal signaling [65,66]. This targeted packaging allows secreted exosomal miRNAs to reprogram recipient stromal, immune, and endothelial cells, enhancing angiogenesis, immune evasion, and tumor progression within the TME.

More recently, hybrid nanocarriers—which combine lipid, polymeric, or inorganic elements—have emerged as multifunctional platforms, often termed theranostic systems. These designs can integrate therapeutic miRNAs and imaging tracers (e.g., fluorescent or radiolabel tags), enabling real-time monitoring of biodistribution and therapeutic effect. Membrane-modified LNPs (coated with cell membranes or targeting ligands) represent one promising direction, offering improved targeting and immune evasion [67]. Such hybrid systems strive to balance biocompatibility, tissue penetration, cargo protection, and controlled release to achieve personalized and precision delivery with minimized systemic toxicity.

Despite encouraging results, delivery efficiency and biodistribution of miRNA-based therapeutics remain inconsistent among formulations and tumor models. In orthotopic HNSCC and other xenograft models, lipid or polymeric nanoparticles typically achieve tumor uptake efficiencies of 5–15% of the injected dose, with the remainder accumulating in liver and spleen because of reticuloendothelial clearance [66,67,68]. PEGylation or ligand-targeting can raise tumor accumulation to ≈20–25%, although intra-tumoral heterogeneity continues to hinder uniform distribution.

Off-target and systemic toxicities have been documented. The anti-miR-122 Miravirsen and the miR-34a mimic MRX34 trials reported dose-dependent immune activation and transient liver enzyme elevation, causing partial trial suspension [69,70]. Comparable preclinical findings in HNSCC and hepatocellular carcinoma models indicated mild hepatic inflammation at high nanoparticle doses without overt cytotoxicity [67,69]. These observations emphasize the need for optimized dosing, controlled release, and targeted biodistribution to ensure therapeutic safety and efficacy.

### 3.3. Clinical Trials and Translational Perspectives

The transition of miRNA-based therapeutics from promising preclinical studies to clinical application in HNSCC is still nascent, but it is progressing. Early clinical experience with miRNA-targeting agents in solid tumors has provided valuable lessons about safety, pharmacokinetics, and delivery feasibility. One of the first compounds to enter human trials was MRX34, a liposomal formulation of a miR-34a mimic. In its Phase I trial in patients with advanced solid tumors, MRX34 demonstrated target engagement and tumor-suppressive activity (with downregulation of BCL2, MET, MYC) at tolerated doses, but the trial was ultimately discontinued due to immune-mediated adverse events [68,69]. Despite the early termination, the dose-dependent modulation of miR-34a targets in circulating cells provided proof-of-concept for miRNA-based therapeutic strategies [70].

However, these early clinical experiences also highlighted critical limitations in the design and evaluation of miRNA-based therapies. Reported clinical setbacks were often associated with systemic immune activation and cytokine release, reflecting insufficient preclinical modeling of immune toxicity and inadequate dose-escalation strategies [65,67]. Moreover, the lack of standardized clinical endpoints—such as validated pharmacodynamic biomarkers, tumor response criteria adapted to miRNA modulation, and longitudinal molecular monitoring—limited the interpretability of efficacy data [69]. These observations emphasize the need for more rigorous inclusion criteria, immune monitoring, and endpoint harmonization in future miRNA trials, especially in tumor types like HNSCC characterized by pronounced molecular heterogeneity.

Another example, TargomiRs (or MesomiR-1), is a minicell-based delivery system containing miR-16 mimics. In early trials (e.g., in mesothelioma), TargomiRs showed a tolerable safety profile and some disease stabilization, bolstering confidence in targeted miRNA replacement approaches [71]. These trials across different cancers serve as important translational benchmarks.

In HNSCC, preclinical studies targeting miR-21 (via antagonists) or restoring miR-375 mimics have delivered promising results: tumor growth reduction, enhanced radiosensitivity, or increased apoptosis in xenograft models. Some in vitro HNSCC cell line work suggests that inhibiting miR-21 can sensitize tumor cells to cisplatin or radiotherapy by upregulating PDCD4 and PTEN [72]. However, direct clinical trials of miRNA therapeutics in HNSCC remain very limited, partly due to challenges of patient heterogeneity, variable miRNA expression, lack of optimized dosing, and achieving tumor-specific delivery without systemic toxicity [71].

Although most miRNA-based interventions remain at the preclinical stage, several candidates have progressed to early clinical testing, providing valuable insights into their safety and translational feasibility. Table 5 summarizes the main miRNA therapeutics evaluated in solid tumors, including their delivery platforms, clinical phases, and reported outcomes, offering a perspective on their potential applicability to HNSCC.

To overcome systemic toxicity and improve localization, emerging translational efforts are exploring local delivery routes—such as intratumoral injection, localized delivery scaffolds, or mucoadhesive hydrogels—to achieve high local concentrations of miRNA agents while minimizing off-target exposure [52]. Although the literature in HNSCC is still limited, analogous strategies are being tested in other solid tumors. Additionally, combinatorial strategies—combining miRNA therapeutics with immune checkpoint inhibitors, radiotherapy, or chemotherapy—are gaining traction, as synergistic effects can amplify anticancer responses and potentially lower required miRNA dosages [73]. Advances in next-generation sequencing, multi-omics and AI-based profiling are expected to help stratify patients and identify predictive miRNA signatures, facilitating more personalized and adaptive miRNA therapeutic regimens in HNSCC.

## 4. Diagnostic and Prognostic Potential of miRNAs in HNSCC

Recent investigations underscore the utility of miRNA expression profiles, especially circulating and exosomal miRNAs, as minimally invasive biomarkers in head and neck squamous cell carcinoma (HNSCC). These biomarkers offer promise for early detection, longitudinal disease monitoring, and prediction of therapy response.

Multiple meta-analyses and cohorts consistently associate miR-21 with worse OS in HNSCC [74,75], and circulating levels show diagnostic [75,76] promise as non-invasive markers; however, disease specificity remains a limitation. Another miRNA of prognostic interest is miR-375. A recent systematic review and meta-analysis determined that low miR-375 expression correlates with worse survival outcomes in HNSCC patients, pointing to its value as a prognostic marker [77]. In earlier literature, reduced miR-375 levels were also linked with metastasis and poor outcome in head and neck cancers [78].

Beyond single miRNAs, multi-miRNA signatures (panels) and predictive models are being developed. For instance, one study constructed an 11-miRNA prognostic signature based on differentially expressed miRNAs from The Cancer Genome Atlas (TCGA), which reliably stratified HNSCC patients according to OS risk [79]. Additional works have proposed miRNA-based prognostic signatures capable of predicting survival more accurately than individual miRNAs [80,81]. While miR-21 is the most extensively profiled, additional miRNAs (e.g., miR-155, miR-146a, miR-375) provide complementary diagnostic/prognostic value and may mitigate disease-specificity concerns when integrated into multi-miRNA panels.

Furthermore, in the non-tissue domain, salivary exosomal miRNAs have shown diagnostic promise for oral and oropharyngeal cancers. A review of salivary exosomal miRs in HNSCC identified several candidate miRNAs (e.g., miR-486-5p, miR-10b-5p, miR-24-3p) as biomarkers for early detection [82].

Taken together, these data indicate that integrating multiple miRNAs into diagnostic or prognostic panels, possibly augmented by machine-learning algorithms, may yield more robust and clinically useful tools for patient stratification and personalized management in HNSCC.

## 5. Challenges and Future Perspectives

Despite significant progress in elucidating the regulatory functions of microRNAs in HNSCC, several major challenges continue to impede their successful clinical translation.

Tumor Heterogeneity and Patient-Specific Targeting. One of the foremost challenges is the intratumoral and interpatient heterogeneity of head and neck cancers. Differences in HPV status, mutational background, and epigenetics drive divergent miRNA profiles and impact therapeutic responses. Indeed, HPV-positive and HPV-negative HNSCC differ in mutation spectra, immune microenvironment, and clinical outcomes, supporting the notion of distinct molecular subtypes [83]. Recent machine-learning based multi-omics analyses in HNSCC have also identified molecular subtypes that correlate with immune infiltration and treatment sensitivity, suggesting that personalized stratification is feasible and necessary [84].

Beyond scientific innovation, several practical and regulatory barriers still limit the clinical translation of miRNA-based therapeutics. Manufacturing costs remain high due to the need for large-scale synthesis of chemically modified oligonucleotides and nanoparticle carriers, as well as the stringent quality control required for good manufacturing practice (GMP) compliance. Moreover, the lack of assay standardization for miRNA quantification and normalization complicates inter-laboratory reproducibility, making multicenter validation difficult. Finally, regulatory pathways for RNA-based drugs are still evolving, with limited harmonization between FDA and EMA guidelines regarding safety assessment and long-term monitoring, which delays clinical adoption [65,67,69].

Off-target risk and delivery limitations. Because miRNAs can regulate many targets, off-target effects are a real concern. However, specific examples in HNSCC are less documented in the literature, so this remains largely conceptual. A more tangible barrier is the poor penetration of nanomedicines in solid tumors due to the TME. Dense extracellular matrix, high interstitial fluid pressure, and abnormal vasculature all contribute to limiting nanoparticle diffusion [85,86].

Combined therapies and theranostic strategies. Translationally, combining miRNA therapeutics with existing modalities is a promising approach. Though direct clinical trials in HNSCC are rare, the principle of multimodal synergy is widely discussed in the nanomedicine/miRNA delivery literature [78]. Theranostic nanoplatforms (i.e., combining therapy and imaging) are a growing field, allowing real-time monitoring of biodistribution and treatment efficacy. Reviews on nanoparticle strategies in cancer therapy mention this integration [87,88,89].

Computational integration and future directions. Artificial intelligence and computational modeling are increasingly used to integrate multi-omics data, infer regulatory networks, and derive patient-specific predictions. The cited multi-omics HNSCC study using machine learning is one such example [83].

To facilitate the clinical advancement of miRNA therapeutics in HNSCC, well-defined experimental design criteria must be adopted. These should include patient stratification according to molecular and immunological signatures (e.g., HPV status, inflammatory cytokine profiles, and miRNA clusters), combination regimens integrating miRNA mimics or inhibitors with immune checkpoint blockade or molecularly targeted therapies, and longitudinal biomarker validation to monitor dynamic changes in circulating miRNAs during treatment. Establishing such standardized frameworks will be crucial for translating preclinical findings into reproducible, regulatory-grade clinical evidence [67,69].

Despite significant advances in understanding miRNA biology, the path toward clinical implementation remains complex. Table 6 presents the major challenges that currently limit the therapeutic use of miRNAs in HNSCC and outlines emerging technological and methodological solutions aimed at improving delivery, specificity, and translational success.

## 6. Conclusions

MicroRNAs have emerged as key molecular regulators that bridge tumor cell biology with the dynamic complexity of the tumor microenvironment in head and neck squamous cell carcinoma. By orchestrating angiogenesis, immune modulation, and stromal remodeling, these small non-coding RNAs exert profound influence on tumor progression and therapeutic resistance. The growing body of evidence supports their dual potential as both diagnostic biomarkers and therapeutic targets, offering a path toward molecularly guided management strategies.

The integration of miRNA-based therapeutics into clinical practice will depend on overcoming current challenges related to delivery specificity, biological stability, and interpatient heterogeneity. Continued advances in nanotechnology, molecular engineering, and computational modeling are expected to refine these approaches, enabling precise and safe modulation of miRNA networks within the tumor milieu. Ultimately, a multidisciplinary framework combining molecular oncology, pharmacogenomics, and systems biology will be crucial for translating miRNA research into personalized and durable therapies for patients with head and neck cancer.

## Figures and Tables

**Table 1:** Key microRNAs involved in tumor–stroma interactions in HNSCC.

microRNA	Expression Pattern	Primary Targets	Molecular Pathways	Functional Effect	Fold-Change/Clinical Correlation
miR-21	Upregulated	PTEN, PDCD4, TPM1	PI3K/AKT, MAPK	Promotes proliferation, invasion, chemoresistance [15,16,17]	~3.5× upregulated in HNSCC tissue; associated with cisplatin resistance and poor prognosis [15,16,17]
miR-31	Upregulated	FIH-1, RhoA	HIF-1, ERK/MMP9	Induces hypoxia adaptation, EMT [18,19]	~2.8× upregulated; linked to metastatic potential in oral SCC [18,19]
miR-34a	Downregulated	MET, Snail, ZEB1	EMT suppression	Inhibits migration, enhances apoptosis [20]	Frequently downregulated in TP53-mutant HNSCC; restoration reduces MET signaling [20]
miR-200c	Downregulated	ZEB1, ZEB2	EMT regulation	Maintains epithelial phenotype [21,22]	Downregulated in invasive HNSCC; associated with stemness via BMI1 [22]
miR-375	Downregulated	JAK2, IGF1R	JAK/STAT, IGF1	Reduces proliferation [23,24]	Among most suppressed miRNAs in HNSCC; inhibits clonogenic growth [23,24]

**Table 2:** Immunoregulatory microRNAs shaping the immune landscape in HNSCC.

microRNA	Expression in HNSCC	Immune Targets	Affected Immune Cells	Functional Role	Fold-Change/Clinical Correlation
miR-146a	Upregulated	IRAK1, TRAF6	Macrophages, epithelial cells	Reduces NF-κB activation, immunosuppression [25,26,27]	Upregulated in tumor tissue; correlates with reduced IL-6/TNF-α secretion [25,26,27]
miR-155	Upregulated	SHIP1, SOCS1	T cells, TAMs	Enhances chronic inflammation, T-cell dysfunction [28,29]	Elevated in HNSCC; linked to M2 macrophage polarization and poor prognosis [28,29]
miR-34a	Downregulated	MET	T cells, macrophages	Increases Th1, reduces PD-L1^+^ TAMs [31]	Downregulated in HNSCC; restoration increases CD8^+^ T cells and reduces immunosuppression [31]
miR-125b	Downregulated	TNFR2	Tregs, CD8^+^ T cells	Promotes cytotoxicity, reduces Tregs [30,32]	Loss of miR-125b-1 in HNSCC; gain enhances IFN-γ^+^ CD8^+^ T cells [30,32]

**Table 3:** Pro- and anti-angiogenic microRNAs regulating vascular and stromal remodeling in HNSCC.

microRNA	Effect	Primary Targets	Process	Outcome	Fold-Change/Clinical Correlation
miR-210	Pro-angiogenic	EFNA3, ISCU1/2	Endothelial adaptation to hypoxia	Promotes neovascularization [36,37]	Induced under hypoxia via HIF-1α; promotes endothelial survival and tube formation [36,37]
miR-21	Pro-angiogenic	PTEN	Endothelial PI3K/AKT	Promotes angiogenic switching [37,38,39]	Overexpressed in HNSCC; enhances vascularization and ECM degradation [37,38,39]
miR-126	Anti-angiogenic	VEGF, PI3K/AKT	Endothelial signaling	Maintains vessel stability [40,41,42,43]	Downregulated in tumors; loss linked to leaky vasculature and metastasis [40,41,42,43]
miR-29c-3p	Anti-angiogenic	C1QTNF6	ECM remodeling	Inhibits invasion and angiogenesis [44]	Suppressed in HNSCC; restoration reduces proliferation and angiogenesis [44]

**Table 4:** Representative miRNA-based therapeutic strategies and preclinical outcomes.

Strategy	miRNA Targeted	Type (Mimic/Inhibitor)	Cancer Model	Key Findings
MRX34	miR-34a (mimic)	Mimic (liposomal)	Multiple solid tumors	Inhibited BCL2, MET; early trial discontinued [46,47,48,49,50,51,52,53,54,55,56,57,58,59,60]
FM-miR-34a	miR-34a	Chemically modified mimic	Prostate cancer	Reduced MYC, MET, AR [56]
Anti-miR-21 (LNA)	miR-21	Inhibitor	Colon and melanoma	Restored PTEN, decreased invasion [57,58,59]
miR-375 mimic	miR-375	Mimic	Preclinical HNSCC	Decreased proliferation via JAK2/IGF1R [23,24]

**Table 5:** Clinical and translational status of miRNA-based therapeutics in solid tumors. AEs—Adverse Events.

Therapeutic Agent	miRNA Target	Delivery System	Cancer Type	Clinical Phase	Outcome
MRX34	miR-34a	Liposomal	Solid tumors	Phase I	Immune-related AEs, proof-of-concept [68,69,70]
TargomiRs (MesomiR-1)	miR-16	Bacterial minicell	Mesothelioma	Phase I	Disease stabilization [71]
Anti–miR-21	miR-21	LNA oligo	Preclinical (HNSCC)	—	Reduced proliferation, enhanced radiosensitivity [72]

**Table 6:** Major barriers and prospective solutions in miRNA-based therapy for HNSCC.

Challenge	Description	Emerging Solutions
Tumor heterogeneity	Variable miRNA profiles in HPV+/− HNSCC	Patient stratification via multi-omics [82,83]
Delivery efficiency	Limited penetration in dense TME	Nanocarriers, exosomes, hydrogels [84,85]
Off-target effects	Multiple mRNA interactions	Sequence optimization, local delivery [86,87]
Clinical translation	Limited trials, immune toxicity	AI-driven biomarker modeling [83,84,85,86,87,88,89]

## Data Availability

No new data were created or analyzed in this study. Data sharing is not applicable to this article.

## Abbreviations

The following abbreviations are used in this manuscript:

HNSCC	Head and Neck Squamous Cell Carcinoma
TME	Tumor Microenvironment
miRNA	MicroRNA
CAFs	Cancer-Associated Fibroblasts
TAMs	Tumor-Associated Macrophages
EVs	Extracellular Vesicles
LNPs	Lipid Nanoparticles
PLGA	Poly(lactic-*co*-glycolic acid)
PEI	Polyethylenimine
PTEN	Phosphatase and Tensin Homolog
NF-κB	Nuclear Factor kappa-light-chain-enhancer of activated B cells
VEGF	Vascular Endothelial Growth Factor
TGF-β	Transforming Growth Factor Beta
MAPK	Mitogen-Activated Protein Kinase
STAT3	Signal Transducer and Activator of Transcription 3
HIF-1α	Hypoxia-Inducible Factor 1-alpha
SMAD	Mothers Against Decapentaplegic Homolog
ECM	Extracellular Matrix
anti-miR	microRNA inhibitor
GMP	Good Manufacturing Practice
